# Effect of terahertz irradiation on DNA damage repair in living cells

**DOI:** 10.1002/2211-5463.70293

**Published:** 2026-06-18

**Authors:** Yuya Ueno, Shota Yamazaki, Hiromichi Hoshina, Masahiko Harata

**Affiliations:** ^1^ Laboratory of Molecular Biochemistry, Graduate School of Agricultural Science Tohoku University Sendai Miyagi Japan; ^2^ RIKEN, Center for Advanced Photonics Sendai Miyagi Japan

**Keywords:** DNA damage repair, DNA double‐strand break, THz irradiation

## Abstract

Various external and internal factors constantly induce DNA damage, with DNA double‐strand breaks (DSBs) being the most severe form of damage. DSBs must be repaired rapidly, and failure to repair leads to the development of cancer and other diseases. Here, we show that terahertz (THz) irradiation decreases the amount of DSBs in a frequency‐dependent manner. Furthermore, DSBs were increased when samples were heated, indicating that the decrease in DSBs by THz irradiation is a nonthermal effect. The modulation of DNA damage repair by THz irradiation provides a noninvasive method with potential medical applications, such as the prevention and suppression of diseases caused by genomic instability.

## Graphical abstract

We investigated the effect of terahertz (THz) wave irradiation on DNA double‐strand break (DSB) repair in living cells. We found that THz irradiation enhanced DSB repair at specific frequencies, whereas heat treatment inhibited it, indicating that this effect is nonthermal and frequency‐specific. Our findings suggest the potential of THz technologies for medical applications.

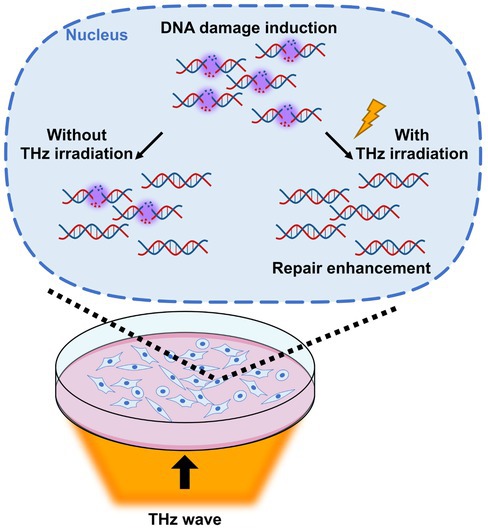

**Abbreviations**
DSB: DNA double‐strand break
THz: terahertz

In addition to the transcription of genetic information and replication during cell growth, DNA damage repair is essential for maintaining genomic integrity. DNA is constantly damaged by exogenous and endogenous factors, including ultraviolet radiation, ionizing radiation, toxins, reactive oxygen species, and heat shock. DNA double‐strand breaks (DSBs) are the most dangerous form of DNA damage and require rapid repair. Errors or delays in DSB repair can lead to genomic instability, including chromosomal deletions and translocations. This instability can lead to cancer, neurodegenerative disorders, and aging [1]. Therefore, enhancing DSB repair may help prevent or inhibit disease progression. While invasive chemical methods have been reported to enhance DSB repair in mammalian cells [2, 3], noninvasive methods have not yet been established.

Thus, this study demonstrated a noninvasive method for enhancing DSB repair via terahertz (THz) irradiation. THz waves are defined as the frequency range of 0.1 to 10 THz, located between the microwave and infrared regions in the electromagnetic spectrum. THz waves can easily penetrate materials such as plastics and paper, and due to their low photon energy, they are incapable of ionizing atoms or molecules [4, 5]. Driven by these properties and recent technological advancements, THz technology is expected to lead to a variety of applications, from next‐generation wireless communications to advanced sensing [5, 6, 7]. Consequently, the biological safety and potential effects of THz wave exposure on living organisms have attracted attention. When THz waves are absorbed by living organisms, they excite the vibrations of biomolecules and surrounding water molecules. This leads to structural changes in these molecules [8, 9]. While it has been widely reported that THz irradiation has biological effects [4, 10], these effects are often attributed to thermal effects due to the strong absorption of THz photons by water. However, several studies have revealed that thermal effects cannot explain the responses of some biological systems to THz irradiation. For example, we found that THz irradiation induces the order–disorder phase transition of cell membrane lipids [11]. We also found that continuous‐wave THz irradiation at 0.46 THz enhanced the polymerization of purified actin protein via a nonthermal effect [12]. The cellular process of cytokinesis is regulated by polymerization and depolymerization of actin, and we showed that THz irradiation affects cytokinesis [13]. One possible molecular mechanism for these nonthermal effects is the alteration of hydrogen‐bonding networks, which may affect protein folding and function. Additionally, THz waves may modulate specific molecular interactions, thus affecting their structural organization.

Actin in the cell nucleus has various functions, in addition to its roles in the cytoplasm. For example, nuclear actin polymerization regulates DNA function and metabolism, including DSB repair [14, 15, 16]. Given our previous findings that THz irradiation enhances actin polymerization, we hypothesized that it may affect DSB repair. In this study, we showed that THz irradiation decreased chemically induced DSBs in a frequency‐dependent manner via a nonthermal effect. These results suggest that THz irradiation noninvasively accelerates DSB repair, thereby indicating its potential for medical applications.

## Materials and methods

### THz irradiation

Continuous‐wave THz radiation was generated using an IMPATT‐diode (TeraSense, San Jose, CA, USA) with frequencies of 0.10 THz, 0.28 THz, and 0.29 THz through the horn antenna. In the 0.10 THz setup, THz radiation was emitted through a 5‐mm‐diameter aperture. We measured the output power using a pyroelectric detector (THz20; Sensor‐und Lasertechnik, Wildau, Germany), which was calibrated at 1.4 THz. In this setup, the output power was 39.5 mW and the power density was 200 mW/cm^2^. In the 0.28 THz and 0.29 THz setups, the THz radiation was focused using a plastic lens and set to a power density similar to that of the 0.10 THz source. In these setups, the output power was 11.5 mW. The Gaussian beam profile was measured with a THz camera (RIGI‐THz‐S2‐Ex; Swiss Terahertz, Zurich, Switzerland), and its full width at half maximum was 2 mm. The power density was 250 mW/cm^2^. THz waves are strongly absorbed by water; therefore, intracellular temperatures were expected to increase. To measure water temperature at the surface under THz irradiation, we used an ultra‐fine sheathed thermocouple (Nippon NetsuDenki SeisakuSho, Tokyo, Japan) with an outer sheath diameter of 0.25 mm. The water temperature increased by 3 °C after 10 min of irradiation at 0.10 THz, 0.28 THz, and 0.29 THz.

Continuous‐wave THz radiation was also generated using a gyrotron (FU CW VIB; University of Fukui, Fukui, Japan) with a frequency of 0.46 THz through a waveguide. In the 0.46 THz setup, the THz wave was generated by a 200‐ms pulse at a repetition rate of 1 Hz, and THz radiation was emitted through a 5‐mm‐diameter aperture. We measured the output power using a pyroelectric detector; the output power was 50 mW, and the peak power density was 250 mW/cm^2^. To measure water temperature at the surface under THz irradiation, we used an ultra‐fine sheathed thermocouple with an outer sheath diameter of 0.01 mm. The water temperature increased by 1 °C.

### Cell culture

HeLa cells (RRID:CVCL_0030) were obtained from the Institute of Development, Aging and Cancer, Tohoku University (Sendai, Japan), which provides authenticated and mycoplasma‐free cells. Once obtained, the cells were propagated, divided into multiple tubes, and frozen to create a working cell bank. For the experiments, the cells were thawed from this bank and used between Passages 3 and 10 to prevent cross‐contamination and mycoplasma infection. The cells were cultured in Dulbecco's modified Eagle's medium (DMEM) (Nacalai Tesque, Kyoto, Japan) supplemented with 10% fetal bovine serum (Gibco; Thermo Fisher Scientific, Waltham, MA, USA) and 100 U/mL penicillin–streptomycin (Thermo Fisher Scientific) at 37 °C in a 5% CO_2_ humidified atmosphere. The cells were seeded in olefin‐based film dishes (Matsunami Glass, Osaka, Japan) and cultured for 24 h to reach 80% confluence.

### DNA damage induction and THz irradiation of HeLa cells

**Fig. 1:**
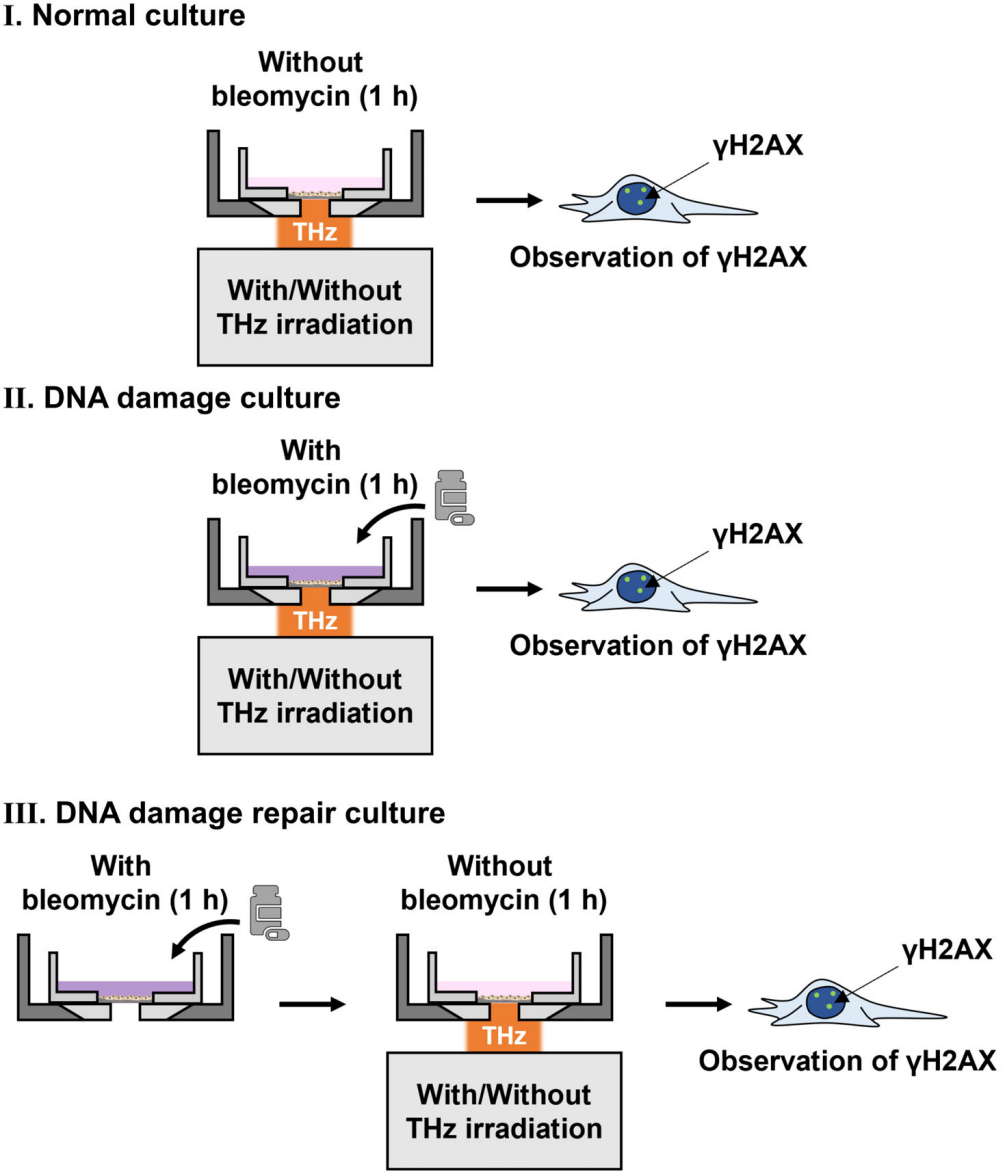
Schematic diagram of cell culture procedures. Experiment I was set as a control to monitor THz irradiation‐induced DNA damage. In Experiment II, bleomycin was included in the culture during the THz irradiation. In Experiment III, bleomycin was removed from the culture before the THz irradiation. Since DNA damage induction and DNA repair occurred concurrently in Experiment II, Experiment III was designed to evaluate the effect of THz irradiation on DNA repair, independent of bleomycin‐induced DNA damage.

HeLa cells were cultured in olefin‐based film dishes (Matsunami Glass, Osaka, Japan), which were set on a stage‐top chamber (BLAST, Kanagawa, Japan) maintained at a temperature of 37 ± 1 °C under a 5% CO_2_ humidified atmosphere. DNA damage was induced via 7.5 μm bleomycin (Tokyo Kasei, Tokyo, Japan) treatment. The irradiation and control experiments were performed on three cultures (Fig. 1). For the normal culture, cells were incubated for 1 h without bleomycin, a DSB inducer, under THz irradiation. For the DNA damage culture, cells were treated with 7.5 μm bleomycin for 1 h to induce DSBs during THz irradiation. For the DNA damage repair culture, cells were treated with 7.5 μm bleomycin for 1 h, washed with fresh medium, and then incubated for 1 h without bleomycin under THz irradiation. Nonirradiated cells were obtained from nonirradiated areas of the same dish. Additionally, we performed a thermal control experiment by heating the sample to 42 °C without THz irradiation.

### Immunofluorescence staining and microscopy

After the THz irradiation experiments, the cells were fixed by treatment with 4% paraformaldehyde in phosphate‐buffered saline (PBS) for 15 min and permeabilized with 0.5% Triton X‐100 in PBS for 10 min at room temperature. Fixed cells were treated with an anti‐phosphoH2AX (Ser139) (DAM1493341; MERCK, Darmstadt, Germany) in 5% bovine serum albumin in PBS for 1 h at 37 °C. After antibody treatment, cells were incubated with Alexa Fluor 488 mouse IgG (A‐11001; Thermo Fisher Scientific) for 1 h at 37 °C. Subsequently, cells were treated with PBS containing 0.2 μg/mL 4′,6‐diamidino‐2‐phenylindole (DAPI) for 5 min at room temperature to stain the nuclei and observed using an IX83 wide‐field fluorescence microscope (Olympus, Tokyo, Japan). Images were captured using an ORCA Flash 4.0 LT PLUS Digital CMOS camera (Model C11440‐42 U30; Hamamatsu Photonics, Shizuoka, Japan). γH2AX foci were counted using the Fiji image analysis software.

### Neutral comet assay

After the THz irradiation experiments, a neutral comet assay was performed using a Comet SCGE assay Kit (Enzo Life Sciences, Farmingdale, NY, USA). 1.0 × 10^4^ HeLa cells were seeded into individual wells of a micro‐Insert 4‐well (ibidi, Madison, WI, USA). After cell cultures were incubated for 24 h, the micro‐Insert 4‐well was gently removed, and THz irradiation was performed as described in the main text. Only the cells in one well of the micro‐Insert 4‐well were irradiated with THz, and the cells were collected by trypsin/EDTA treatment using a cloning ring (RING‐07; IWAKI, Shizuoka, Japan). The cells outside the cloning ring were collected as THz nonirradiated cells by trypsin/EDTA treatment. The cells were resuspended in PBS, combined with the cells and molten LMAgarose in a 1:10 ratio, and pipetted onto Comet Slides. The slides were incubated in prechilled lysis solution for 1 h and electrophoresed in TBE buffer at 35 V for 20 min at 4 °C. The slides were stained with SYBR Gold (Thermo Fisher Scientific) for 30 min and imaged. The Olive tail moment was measured using CometScore 2.0 (TriTek Corp., Sumerduck, VA, USA).

### Statistical analysis

All statistical analyses were performed using the Origin Pro 2025 software. For statistical analyses, three independent experiments were performed. The *P*‐value (*P*) was calculated using Welch's *t*‐test. A *P*‐value of less than 0.05 was defined as a statistically significant difference between two groups.

## Results and Discussion

We observed H2AX (γH2AX) phosphorylation as the DSB marker in HeLa cells with and without THz irradiation. Formation of γH2AX is an early cellular response to DSB induction, and γH2AX quantification is a well‐established method for detecting DSBs [17]. The irradiation and control experiments were performed on three cultures: normal culture, DNA damage culture, and DNA damage repair culture (Fig. 1; details in the Materials and Methods section). Additionally, because THz irradiation increases the temperature of the samples by a few degrees, thermal control experiments were performed by heating the samples to 42 °C.

**Fig. 2:**
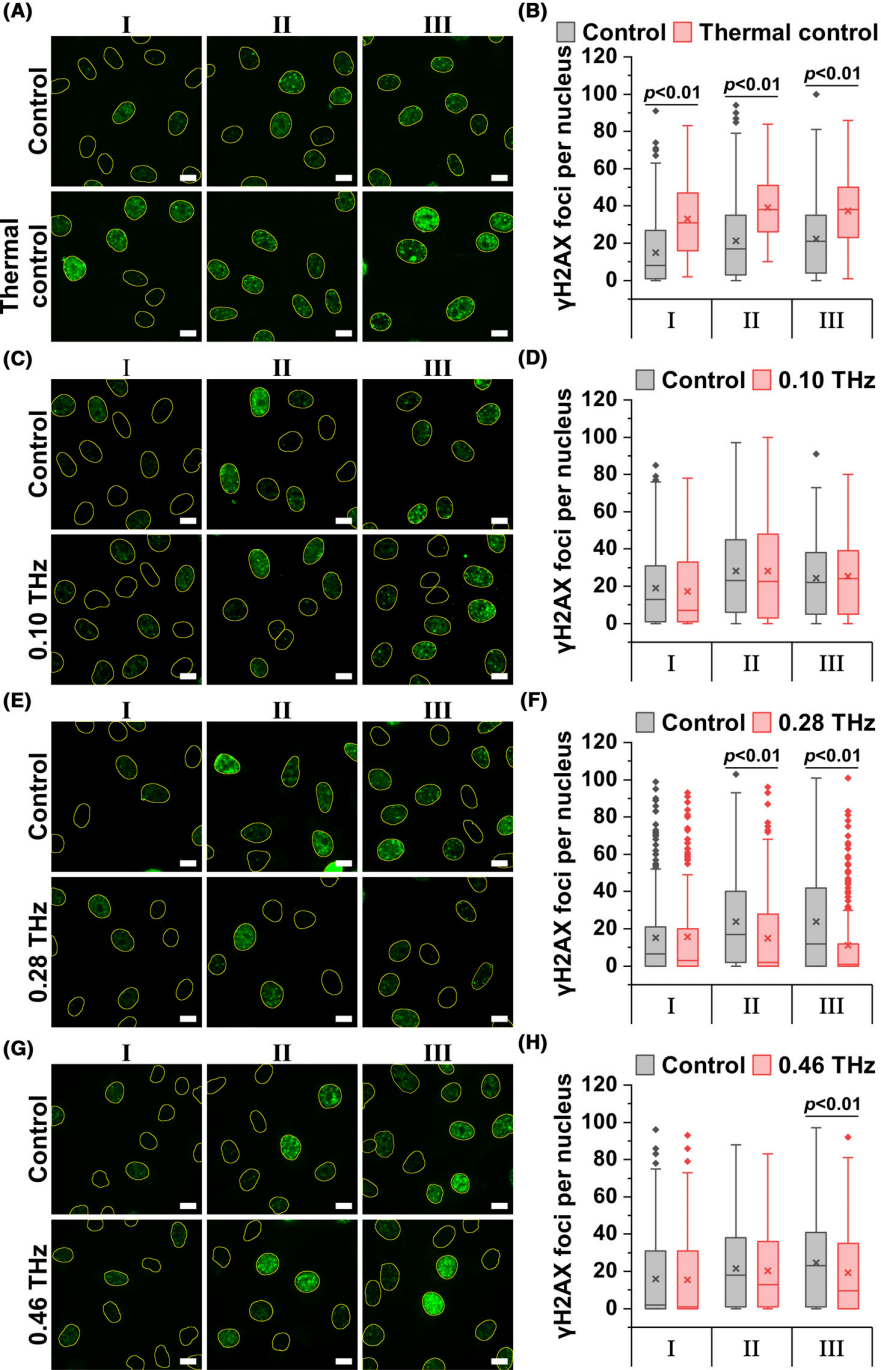
Effects of THz irradiation on the amount of γH2AX. (A, C, E, G) Fluorescence microscopy images of HeLa cells stained with a γH2AX antibody. The nuclei are outlined in yellow. Scale bar = 10 μm. (B, D, F, H) The γH2AX foci per nucleus. Images and γH2AX foci were measured without bleomycin treatment (I), 1 h after bleomycin treatment (II), and a 1 h culture after bleomycin treatment and subsequent removal (III). The samples were measured without THz irradiation (control), at 42 °C without THz irradiation (A, B), with 0.10 THz irradiation (C, D), with 0.28 THz irradiation (E, F) and with 0.46 THz irradiation (G, H). Data from three independent experiments are presented as a box plot. The horizontal line within each box indicates the median value, and the mean value is indicated by a cross, while the box boundaries represent the interquartile range (25th–75th percentiles). The whiskers extend to the minimum and maximum values within 1.5 times the interquartile range, and individual points beyond this range correspond to outliers plotted as diamonds. In each experiment, between 45 and 141 cells were measured per condition. Statistical significance was calculated using Welch's *t*‐tests.

Fig. 2A,B shows the thermal control experiments. Fluorescence microscopy images of γH2AX in the nuclei of HeLa cells cultured at 37 °C and 42 °C (Fig. 2A), and the quantified γH2AX foci per nucleus (Fig. 2B) are shown. The number of γH2AX foci increased in all procedures under the thermal controls, indicating that heating HeLa cells by 5 °C induces DSBs and inhibits their repair, consistent with previous reports [18, 19].

Fig. 2C,D compares samples irradiated at 0.10 THz with the control samples. No significant difference was observed, suggesting that irradiation does not induce DSBs under these conditions, as previously reported [20, 21].

Fig. 2E,F presents the samples irradiated at 0.28 THz. When the cells were incubated without bleomycin, no significant difference was observed (2F‐I). In contrast, the γH2AX foci in the bleomycin‐treated samples decreased after irradiation (2F‐II), a condition in which DSB induction and repair proceed simultaneously, suggesting that THz irradiation either inhibits γH2AX focus formation or promotes DSB repair. Moreover, the γH2AX foci decreased drastically upon THz irradiation and bleomycin removal (2F‐III), a condition in which the DSB repair process is predominant and new DSBs are less likely to be introduced, strongly suggesting that THz irradiation promotes DSB repair.

The similar effect of THz irradiation on the reduction in γH2AX foci was also observed at 0.46 THz, but to a lesser extent (Fig. 2G,H). When the cells were incubated without bleomycin, no significant difference was observed after irradiation (2H‐I). In bleomycin‐treated samples (2H‐II and III), the γH2AX foci decreased significantly only upon THz irradiation and bleomycin removal (2H‐III).

These results suggest that 0.28 THz and 0.46 THz irradiation do not cause new DSBs and, to varying degrees, enhance the repair of DSBs induced by bleomycin. Together with the lack of effect of 0.1 THz irradiation on DSB repair, this phenomenon is shown to be frequency dependent. Notably, since the thermal control had the opposite effect, these results suggest that irradiation at 0.28 and 0.46 THz enhances DSB repair processes through a nonthermal mechanism.

**Fig. 3:**
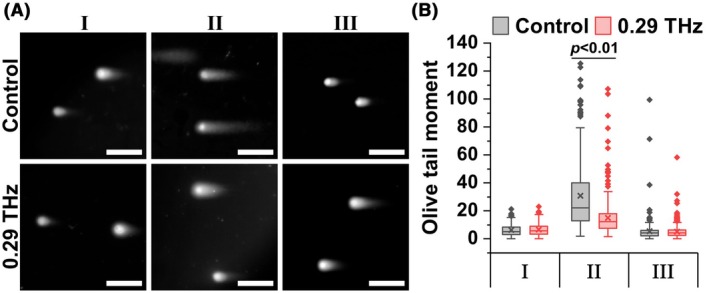
Effects of THz irradiation on DNA damage repair by the Neutral Comet assay. (A) HeLa cells were applied to the Neutral Comet assay. Fluorescence microscopy images of HeLa cells stained with SYBR Gold are shown. Scale bar = 100 μm. (B) The Olive tail moment was measured using CometScore 2.0. The Olive tail moment of cells without bleomycin treatment (I), 1 h after bleomycin treatment (II), and 1 h culture after bleomycin treatment and subsequent removal (III) was analyzed. The samples were measured without THz irradiation (control) and with 0.29 THz irradiation. Data from three independent experiments are presented as a box plot. The horizontal line within each box indicates the median value, and the mean value is indicated by a cross, while the box boundaries represent the interquartile range (25th‐75th percentiles). The whiskers extend to the minimum and maximum values within 1.5 times the interquartile range, and individual points beyond this range correspond to outliers plotted as diamonds. In each experiment, between 49 and 153 cells were measured per condition. Statistical significance was calculated using Welch's *t*‐tests.

We also evaluated the Olive tail moment in the comet assay, a highly sensitive method for measuring DSBs. In this assay, DSBs are specifically detected by neutral electrophoresis and quantified by measuring the Olive tail moment [22, 23]. Fig. 3A,B presents the samples irradiated at 0.29 THz. Fluorescence microscopy images of comet formation on HeLa cells cultured with or without THz irradiation (Fig. 3A), and the quantified Olive tail moment (Fig. 3B) are shown. No significant differences were observed when cells were incubated without bleomycin (3B‐I). This is consistent with the γH2AX results (2F‐I) and suggests that THz irradiation does not cause DSBs. Moreover, the Olive tail moment of the bleomycin‐treated samples decreased after irradiation (3B‐II). This is consistent with the γH2AX results (2F‐II) and suggests that THz irradiation enhances DSB repair processes. In contrast, there were no significant differences in the bleomycin removal conditions (3B‐III) because most of the induced DSBs were repaired. However, since it has been reported that γH2AX does not disappear immediately after DSB repair is completed [24, 25], the decrease of γH2AX by THz irradiation in this condition (2F‐III) further supports the hypothesis that THz irradiation enhances DSB repair.

In this study, we showed that irradiation at 0.28, 0.29, and 0.46 THz reduces the number of DSBs, suggesting that THz irradiation enhances DSB repair. However, this effect was not observed at 0.10 THz irradiation, indicating its frequency‐dependency. Furthermore, thermal control experiments at 42 °C showed no reduction in DSBs, indicating that irradiation at 0.28, 0.29, and 0.46 THz enhances DSB repair processes via a nonthermal effect. Although the mechanism is still unclear and further analysis is required, we propose two possibilities.

The first possibility involves increased nuclear actin polymerization. We previously reported that irradiation at 0.28 and 0.46 THz promotes actin polymerization in living cells [13]. This effect likely occurs within the nucleus, and the resulting increase in nuclear actin polymerization could accelerate DSB repair. As reported in previous studies, nuclear actin contributes to the clustering of damage sites or their relocation to the nuclear periphery, thereby enhancing repair efficiency [26, 27].

The second possibility involves modulating chromatin accessibility. Chromatin accessibility of nuclear proteins regulates genome functions, including DNA repair [28]. Importantly, THz irradiation has been reported to affect histone modification [29] and chromatin accessibility [30, 31]. Alteration of chromatin structure or histone modification by THz irradiation may accelerate the recruitment of repair factors to DSB sites, thereby enhancing DSB repair.

In either case, unlike irradiation at 0.10 THz, irradiation at 0.28 THz and 0.46 THz may effectively excite the vibrations of biomolecules and surrounding water molecules involved in DSB repair. It is expected that molecular vibrations are differently affected by THz irradiation frequency and that THz irradiation thereby accelerates DSB repair in a frequency‐dependent manner.

In conclusion, our observations suggest that THz irradiation enhances DSB repair. This effect is the opposite of that of heat; moreover, it is frequency‐dependent. Our findings provide a novel, noninvasive method for enhancing DSB repair without drugs, suggesting potential medical applications for THz irradiation. This includes preventing and suppressing diseases caused by genomic instability, such as cancer and neurodegenerative diseases. Recent developments in THz technologies have produced high‐output, compact, and affordable radiation sources with various proposed applications, such as 6G wireless communication [5, 6, 7]. Our study provides a novel perspective on the use of these THz technologies.

## Conflict of interest

The authors declare no competing interests.

## Author contributions

YU, SY, and MH conceived the experiment. YU performed all the experiments and data analysis. YU and MH wrote the manuscript. SY and HH designed the experimental setup and modified the manuscript. All authors contributed to analysis and interpretation of data.

## Data Availability

The data that support the findings of this study are available from the corresponding author upon reasonable request.
